# Nano-sized Superlattice Clusters Created by Oxygen Ordering in Mechanically Alloyed Fe Alloys

**DOI:** 10.1038/srep11772

**Published:** 2015-07-02

**Authors:** Yong-Jie Hu, Jing Li, Kristopher A. Darling, William Y. Wang, Brian K. VanLeeuwen, Xuan L. Liu, Laszlo J. Kecskes, Elizabeth C. Dickey, Zi-Kui Liu

**Affiliations:** 1 Department of Materials Science and Engineering, The Pennsylvania State University, University Park, PA 16802, USA; 2 Department of Materials Science and Engineering, North Carolina State University, Raleigh, NC 27695, USA; 3 U.S. Army Research Laboratory, Weapons and Materials Research Directorate, RDRL-WMM-F, Aberdeen Proving Ground, MD 21005, USA

## Abstract

Creating and maintaining precipitates coherent with the host matrix, under service conditions is one of the most effective approaches for successful development of alloys for high temperature applications; prominent examples include Ni- and Co-based superalloys and Al alloys. While ferritic alloys are among the most important structural engineering alloys in our society, no reliable coherent precipitates stable at high temperatures have been found for these alloys. Here we report discovery of a new, nano-sized superlattice (NSS) phase in ball-milled Fe alloys, which maintains coherency with the BCC matrix up to at least 913 °C. Different from other precipitates in ferritic alloys, this NSS phase is created by oxygen-ordering in the BCC Fe matrix. It is proposed that this phase has a chemistry of Fe_3_O and a D0_3_ crystal structure and becomes more stable with the addition of Zr. These nano-sized coherent precipitates effectively double the strength of the BCC matrix above that provided by grain size reduction alone. This discovery provides a new opportunity for developing high-strength ferritic alloys for high temperature applications.

The ever-increasing demand for higher energy efficiency continuously pushes the properties of structural alloys towards greater strength, higher toughness, and better resistance to high temperature environments. For example, the applications of ferritic alloys, one of the most important structural engineering alloys in our society, in extremely harsh environments such as high-pressure steam pipes, heat exchangers, and nuclear reactors necessitate a new generation of alloy chemistries whose properties meet the demands of these environments^1^,^2^. It has been well established that one of the most effective strengthening mechanisms in alloys is to create and maintain, under service conditions, coherent interfaces between the alloy matrix and its precipitates^3^. The precipitates can be either thermodynamically stable or meta-stable.

The successful cases include age-hardened Al-based alloys and Ni-based superalloys. For Al-based alloys, the coherent precipitates include the metastable Guinier-Preston zones and a series of metastable phases, while for Ni-based superalloys, it is the stable L1_2_ precipitates, both within the FCC matrix^4^,^5^,^6^,^7^; the Co-based superalloys under development adhere to the same mechanisms^8^. The full coherency between the precipitates and matrix leads to strengthening by mechanisms such as the creation of antiphase boundaries (only for ordered superlattice precipitates, such as the L1_2_ phase in Ni-based superalloys) or coherency strains that impede dislocation motion^3^,^5^. More importantly, coherency gives rise to low interfacial energies between the precipitates and matrix, thus increasing thermal stability by significantly reducing coarsening^9^ or the transitioning to incoherency, i.e., over aging^10^. As a result, these strengthened alloys are known to preserve their excellent mechanical properties at elevated temperatures.

In ferritic alloys, BCC-Cu precipitates in the Fe-Cu system and β’ (NiAl-type) precipitates in the Fe-Ni-Al system have been explored via aging due to their lattice coherency with the BCC α-Fe matrix. Unfortunately, it was found that BCC-Cu easily transforms to FCC at 550 °C, inducing harmful brittleness^11^. Likewise, the rapid decrease of yield strength around 600 °C and high coarsening rates impede the application of NiAl-strengthened ferritic alloys at high temperatures^12^,^13^. In contrast to conventional aging methods, mechanical alloying provides alternative possibilities in the search and creation of precipitates, because of its capability to synthesize highly supersaturated solid solutions and metastable intermetallics^14^,^15^. It may be noted that these metastable phases may not be accessible from equilibrium processing routes. One example is the oxide dispersion strengthened (ODS) steels produced through mechanical alloying of an alloy and oxide powders^16^,^17^. The subsequent reactions between the alloy and oxide result in the formation of complex nanoclusters and nanoparticles, whose atomic structures are intriguing and being actively investigated^16^,^18^.

In the present work, we report the discovery of a new, oxygen-enriched, nano-sized superlattice (NSS) clusters (2–5 nm) in ball-milled Fe and Fe alloys which maintain coherency and fine sizes up to at least 913 °C, much higher than the stability temperature of known coherent precipitates in ferritic alloys^11^,^12^. Being fully coherent with the α-Fe matrix, the NSS clusters have given rise to an additional strengthening for these Fe alloys, likely similar in nature to the aforementioned Ni- and Al-based alloys. Contrary to other precipitates and dispersion particles in ferritic alloys and ODS steels that need additional alloying elements or oxides, the NSS clusters are created by oxygen ordering in α-Fe BCC lattice and are further stabilized by the addition of Zr. The discovery of this new phase reveals a new opportunity for developing high-strength nanostructured ferritic alloys for extreme environments.

## Results and Discussion

**Figure 1:**
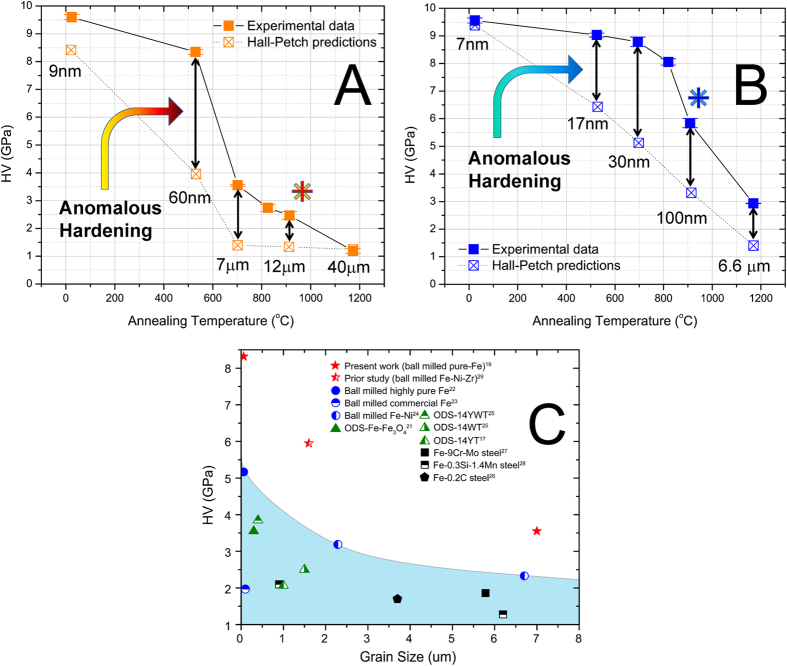
Indentation hardness of (**A**) unalloyed Fe (orange) and (**B**) Fe-1at.% Zr (blue) versus annealing temperature for 1 hour annealing time. The solid squares indicate the observed hardness and the open squares indicate the hardness predicted by the Hall-Petch relationship for ball-milled Fe given by Jang and Koch^20^. Hardness and grain size data are by Darling *et al.*^19^. The asterisk (*) indicates the 913 °C annealed samples analyzed by TEM in the present work. (**C**) Hardness comparison between the samples in the present work, our prior study and some other strengthened ferritic alloys with similar grain-size: markers in red (unalloyed Fe samples in present work and prior study^19^,^29^); markers in blue (ball-milled Fe-alloys^22^,^23^,^24^); markers in green (ODS^17^,^21^,^25^); markers in black (solute and precipitates strengthened steels^26^,^27^,^28^).

The Vickers microhardness versus annealing temperature of the high-energy ball milled Fe (unalloyed Fe) and Fe-1at.% Zr samples, measured in our prior studies^19^, is shown in Fig. 1A,B. It is commonly accepted that the hardness of these alloys is dominated by the effects of grain boundary strengthening^19^. However, the substantial hardness difference between the Hall-Petch predictions for the ball milled α-Fe^20^ and the experimental observations indicate that there should be additional mechanisms at play. This is particularly evident for samples that have been annealed between 530 and 913 °C. At a grain size of 7 μm, the hardness of the unalloyed Fe sample is close to three times of the value predicted based on the Hall-Petch relation (Fig. 1A). This particular strengthening phenomenon can be preserved to some extent even after annealing at 1173 °C for one hour (Fig. 1B) in the Fe-1at.% Zr sample. Such an observation signifies that this strengthening phenomenon is enhanced by alloying with Zr and persists despite being exposed to high homologous temperatures, thereby displaying a significant increase in thermal resistance. In Fig. 1C, the hardness values of the unalloyed Fe samples annealed at 530 and 700 °C are also compared to some other strengthened ferritic alloys with similar grain sizes^17^,^21^,^22^,^23^,^24^,^25^,^26^,^27^,^28^. Another ball-milled Fe-based alloy in prior a study, prepared in a similar way as the present work, is also included for comparison^29^. As seen, due to this abnormal strengthening phenomenon, samples in the present and prior investigations exhibit significant hardness improvements in the grain-size range of submicro or micrometers. One may notice that the hardness of the Fe-1at.% Zr sample annealed at 913 °C is about 2 GPa lower than that of the unalloyed Fe sample annealed at 530 °C, though their grain-sizes are similar. This could be attributed to the bimodal grain-size distribution of the 1at.% Zr sample, induced by high temperature annealing, which is not present in the unalloyed nanocrystalline Fe annealed at 530 °C^19^.

**Figure 2:**
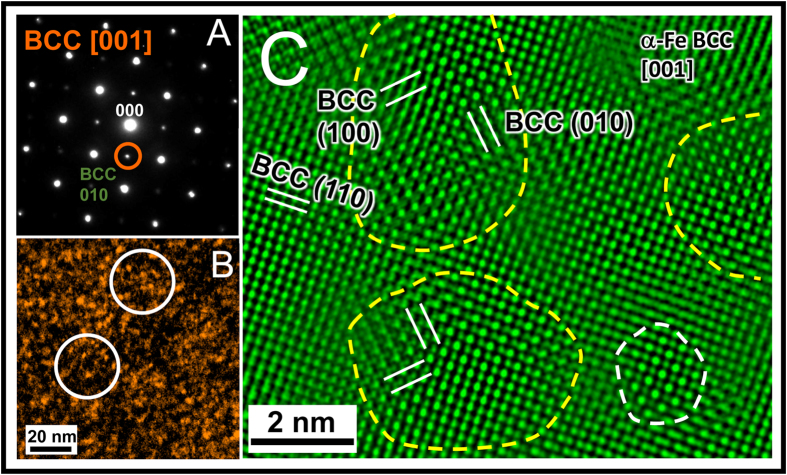
SAED pattern, dark and bright field image of the unalloyed Fe sample. (**A**) SAED pattern by tilting α-Fe to [001] showing {100} superlattice reflections (marked with circle). (**B**) dark-field image using the 010 superlattice reflection marked in (**A**) showing dispersed phase 4.4 ± 0.9 nm in size. (**C**) [001] HRTEM image showing clustering of ordered superlattice phase (2-5 nm in size). Dashed lines mark approximate boundaries of a few clusters.

The microstructures of the unalloyed Fe and Fe-1at.% Zr samples, which were annealed at 913 °C for one hour, were analyzed by transmission electron microscopy (TEM). Energy dispersive x-ray spectroscopy (EDS) and electron energy loss spectroscopy (EELS) were performed on samples for composition analysis. The EELS results revealed that surface oxides existed on both the Fe-1at.%Zr and unalloyed Fe TEM samples, which has the structure of Spinel Fe_3_O_4_ as identified by selected area electron diffraction (SAED); see Supplementary Section 4 for more details. Further scrutinizing the SAED patterns of the α-Fe matrix, e.g., Fig. 2A (unalloyed Fe sample), reveals an additional set of reflections (marked with an orange circle) corresponding to α-Fe forbidden {100} reflections^30^. In fact, the presence of these α-Fe {100} forbidden reflections are consistently observed in the α-Fe <100> SAED patterns for both unalloyed Fe and Fe-1at.% Zr samples. Given the existence of the surface spinel Fe-oxides, it is important to note that the observed α-Fe {100} forbidden reflections are a bulk phenomenon and cannot be attributed to surface Fe oxides (see Supplementary section 5). These additional reflections also do not match the structure of any other existing Fe oxides. They are believed to have come from a superlattice ordering in the α-Fe matrix as the ordering breaks the translational symmetry of the BCC crystal lattice (or it can be equivalently thought of as a doubling of the supercell dimensions)^30^,^31^. By using the {100} forbidden reflections in Fig. 2A to obtain a TEM dark field (DF) image, the morphology, size, and distribution of the superlattice regions reveal a random dispersion of equiaxed nano-clusters with a size range of 2–5 nm, as shown in Fig. 2B. The spatial dispersion of the clusters is at the same length scale. Fig. 2C shows the high resolution TEM (HRTEM) image of the unalloyed Fe sample along the [001] zone-axis. It is clearly shown that the nano-clusters have a perfect ordered superlattice structure in the α-Fe matrix. A dispersed superlattice phase with similar size and distribution was also observed in the Fe-1at.% Zr sample; see Fig. S3A for the representative SAED pattern and Fig. S3B for a corresponding DF-TEM image of the morphology and distribution.

**Figure 3:**
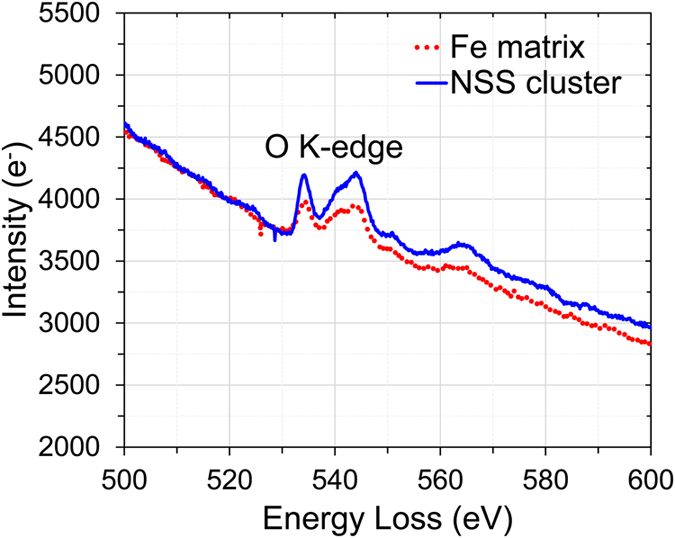
Comparison of the EELS O K-edge spectra from an NSS cluster (blue solid line) and the adjacent α-Fe matrix (red dash line) in the unalloyed Fe sample, revealing the oxygen enrichment of the NSS cluster.

**Figure 4:**
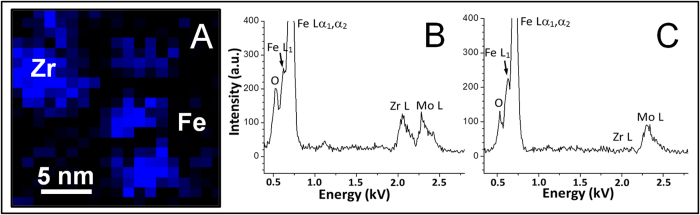
(**A**) Zr elemental map in a grain of the Fe-1at.% Zr alloy recorded in STEM/EDS mode (probe size: 1 nm) showing Zr-enriched clusters with a size of ~5 nm. (**B**) EDS spectrum from Zr-enriched cluster showing a high oxygen content. (**C**) EDS spectrum from Fe matrix showing a lower oxygen content and no Zr.

To further investigate the chemistry of the NSS clusters in the ball-milled Fe sample, EELS was performed. The nano-sized TEM beam was placed on the individual NSS clusters and on the surrounding α-Fe matrix (as shown in Fig. 2C), respectively. Fig. 3 shows a comparison of the EELS spectra for the O K-edge from the NSS clusters and from the adjacent α-Fe matrix. It is seen that the net intensity of the O K-edge of the NSS clusters is evidently higher than that of the adjacent α-Fe matrix, revealing O-enrichment in these NSS clusters. The chemistry of the NSS clusters in the Fe-1at.% Zr sample is also investigated. A Zr elemental map in the Fe-1at.% Zr sample, recorded in STEM/EDS (Scanning Transmission Electron Microscopy/Energy Dispersive X-ray Spectroscopy) mode with a probe size of 1 nm, is presented in Fig. 4A. The blue regions (Zr-enriched) are found to be about 5 nm, the same as the size of the NSS clusters revealed by a dark-field image in Fig. S3B, which indicates enrichment of Zr in the clusters. Fig. 4B,C show EDS spectra from a Zr-enriched cluster (higher Zr-L peak) and the Fe matrix (almost no Zr-L peak), respectively. Based on the EDS analysis using Zr-K and Fe-K peaks (not shown), the concentration of Zr in the NSS clusters is around 6.0 at.%, substantially higher than the equilibrium solubility of Zr in α-Fe^32^. Furthermore, the intensity of oxygen in Fig. 4B is nearly two times higher than that in Fig. 4C, indicating that oxygen is also enriched in the NSS clusters for the Fe-1at.% Zr sample. Further composition analysis, in terms of other possible impurity elements, is provided in supplementary section 2. By excluding the possibility of other impurities in the α-Fe matrix, it is concluded that the NSS cluster is mainly composed of Fe and O.

Since no oxide powders are added to the alloy as in ODS steels, it is of interest to ascertain the source of the oxygen in the NSS clusters. The Fe powders, before ball milling, were analyzed by TEM. It was found that a mixed Fe-oxide layer is present on the surface of the starting Fe powders. Because no Fe-oxides are found in the ball-milled samples (Fig. S1), it is suggested that these pre-existing surface Fe oxides were incorporated into the α-Fe matrix during the high-energy ball milling process, providing the source of the oxygen in the α-Fe matrix to form the O-enriched NSS clusters. Additionally, shown in Fig. S7 is a typical [001]_α-Fe_ SAED pattern for a grain in the starting Fe-powder. As expected, no {100} superlattice reflections are present. This also confirms the contributing effect of high-energy ball milling on formation of the ordered NSS clusters. Prior literature supports these claims. During high energy mechanical alloying, it has been shown that oxides can be decomposed, and their constituents forcibly dissolved into the Fe matrix, providing the oxygen source to form oxide nanoparticles. The initial oxides can either be from surface oxidation on the alloying powders or outside additions^17^,^33^. The extent of dissolution remains an active area of current research.

**Figure 5:**
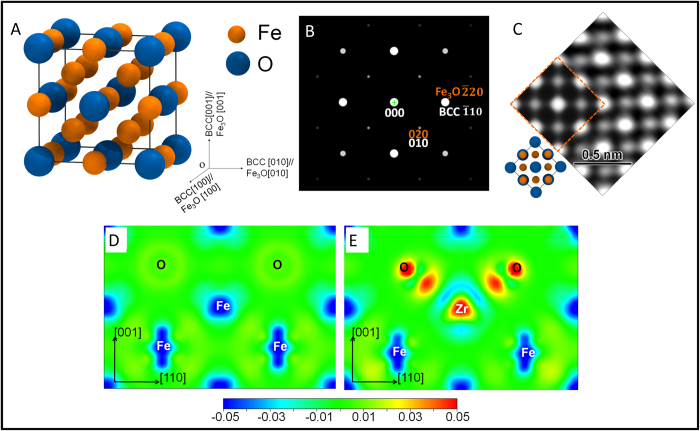
(**A**) Proposed Fe_3_O unit cell [D0_3_ (A_3_B) structure)], which contains 12 Fe atoms and 4 O atoms and has a space group symmetry of Fm
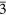
m. The inset shows orientation relationship between Fe_3_O unit cell and α-Fe unit cell, i.e. α-Fe [100]// Fe_3_O [100], α-Fe [010]// Fe_3_O [010], α-Fe [001]// Fe_3_O [001]. (**B**) Simulated [001] diffraction pattern of the proposed Fe_3_O structure (here Fe_3_O {020} reflections correspond to BCC {010} superlattice reflections). (**C**) Experimentally observed [001] HRTEM image (unalloyed Fe sample) compared with the simulated HRTEM image of [001] projection of the proposed Fe_3_O structure (inset within an orange dotted box), showing good agreement. The [001] structure projection of Fe_3_O is shown in corner (left bottom). (**D**) Differential charge density map of the Fe_3_O unit cell in (1
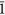
0) plane without Zr. (**E**) Differential charge density map of Fe_3_O unit cell in (1
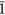
0) plane with one Fe atom replaced by Zr. The unit of charge density is e/Å^3^.

The superlattice reflections are observed in both unalloyed Fe and Fe-1at.% Zr samples; as such, the NSS clusters are more likely to arise from oxygen ordering, rather than other ordering mechanisms, such as Zr ordering. Based on the above observations, we propose the NSS clusters with a D0_3_ (A_3_B) structure, where Fe occupies the A sites and O occupies the B sites to form a Fe_3_O unit cell (Fig. 5A). Similar crystal structure exists in ferritic intermetallics (Fe_3_Al and Fe_3_Si)^34^,^35^ and other BCC metal oxides (Mo_3_O)^36^. The proposed structure model could be thought of as a FCC superlattice ordering in the BCC matrix. The FCC unit cell consists of eight(2 × 2 × 2) α-Fe unit cells, in which the Fe atoms on the corners and the face-centered sites are replaced by O atoms. Fig. 5B shows the simulated [001] diffraction pattern of the Fe_3_O structure. As expected, because of the oxygen ordering, additional superlattice {100} reflections appear on a typical BCC [001] diffraction pattern, which is in good agreement with the observations in Fig. 2A and S3A. Moreover, as shown in Fig. 5C, the simulated Fe_3_O HRTEM image along the [001] zone-axis (inset in Fig. 5C) is consistent with the experimentally observed one in the unalloyed Fe. In the present work, the [001] zone axis is particularly useful and critically important for identifying the ordered structure of NSS clusters because the corresponding reflections cannot be obstructed by neither the double diffraction nor the reflection from surface oxides. Further comparisons between simulated SAED patterns of the proposed Fe_3_O structure and observed patterns of the α-Fe matrix, along other zone-axes, are shown in Fig. S8.

The proposed atomic structure of the NSS cluster does not correspond to any of the known equilibrium Fe oxide phases; therefore, it is further analyzed by atomistic simulations for verification. First-principles calculations based on density functional theory (DFT) are performed to find the equilibrium state of the proposed crystal structure. Three different exchange-correlation functionals are applied to study the proposed structure systematically. In the unrelaxed Fe_3_O cell, the lattice parameters of all three directions were initially set to be twice that of the α-Fe lattice parameter so that the cell would be perfectly coherent with the BCC matrix. After relaxation, the Fe_3_O supercell retains the initial space group symmetry, Fm
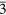
m. The lattice parameter of the relaxed supercell shows an average lattice mismatch of −0.7% when compared to the initially set value, indicating a good coherency with the BCC matrix (Table S2). The formation enthalpy of the proposed Fe_3_O structure with respect to the pure α-Fe and oxygen molecule is also calculated via DFT (Table S4). Although the 0 K enthalpy of formation is as high as 39.0 kJ/mol-atom, it is entirely possible to produce this non-equilibrium oxygen ordering structure at the nano-scale through mechanical alloying, which has been widely used to synthesize non-equilibrium alloy phases^14^,^15^. For example, the meta-stable BCC W-Cu solid solution, which has a positive enthalpy of formation around 35 kJ/mol-atom, was synthesized via mechanical alloying processing^37^. Therefore, we propose that the Fe_3_O NSS cluster arises from metastable FCC oxygen ordering on the parent α-Fe lattice and has a D0_3_ crystal structure.

In addition to elucidating the atomic structure of NSS clusters, it is important to understand the role of Zr in stabilizing these clusters, as Zr is observed to enrich the nano-clusters, and the strengthening induced by the NSS clusters is better preserved in Fe-1at.% Zr samples after high temperature annealing. In the first-principles calculations, one Zr atom is added into the Fe_3_O supercell to replace one Fe atom for the sake of consistency with the experimental composition. The Zr atom prefers to occupy the first nearest neighbor site (FNS) with respect to one O atom based on the data and analysis in the Supplementary Information section. After fully relaxing the supercell, the deformation electron density in the (1
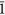
0)-plane, represented by the charge density difference for the fully relaxed structure with and without self-consistent calculations, are plotted in Fig. 5D,E for the Fe_3_O and Fe-O-Zr supercells, respectively. The electron density between Fe and O is low in both figures, indicating that the interactions between them are weak. However, the charges of Zr and O exhibit significant delocalization and polarization along the [111] direction, implying strong bonding between Zr and O. This explains the co-enrichment of Zr and O in the EDS data as Zr and O prefer coupling with each other to form strong chemical bonds.

Considering that the NSS clusters are in a metastable state in Fe-matrix, they start to decompose at high temperatures. As shown in Fig. 1, after annealing at 1173 °C, the hardness enhancement due to the presence of nano-clusters in pure Fe is practically eliminated since the clusters are probably decomposed by annealing. However, because of the strong chemical bonds between Zr and O, the NSS clusters could be further stabilized at higher temperatures by Zr, which are found to enrich in these nano-clusters. As a result, the Fe-1at.% Zr samples can retain the higher-than-predicted hardness even after annealing at high temperatures.

## Conclusions

A metastable Fe_3_O phase, in the form of nano-sized superlattice (NSS) clusters, is produced by mechanical alloying of Fe powders containing surface oxides. Originating from an ordering of oxygen in the BCC Fe-matrix, the Fe_3_O phase is fully coherent with the α-Fe matrix and forms ordered superlattice clusters with sizes on the order of 2 to 5 nm. As a result, a new, nano-scale coherent precipitate strengthening phenomenon results in this ball-milled α-Fe. An enhancement of hardness is observed in the samples with submicro or micrometer grain sizes, without high concentration alloying or the addition of additional oxides, such as yttria or titania, which are commonly used in ODS alloys^16^,^25^,^38^,^39^,^40^,^41^. These D0_3_-Fe_3_O nanoparticles maintain coherency with the α-Fe matrix up to annealing temperatures of 913 °C. The thermal resistance of the strengthening effects caused by this nanoparticle can be enhanced by alloying with Zr to even higher temperatures. With such advantages of high strength and heat resistance, this type of superlattice-precipitate-strengthened Fe alloy reveals a new opportunity to develop high-strength nanostructured ferritic alloys for high temperature applications.

## Method

### Sample Preparation

99at.% Fe-1at.% Zr was mechanically alloyed from elemental powders via high-energy ball milling in a SPEX 8000 shaker mill. Milling vials were carefully prepared in an ultra-high purity argon atmosphere to avoid excessive oxygen contamination. Milling was performed for 24 hours with a 10:1 ball to powder mass ratio. Control samples with no added Zr were also prepared to understand the role of Zr in the hardening and microstructural evolution. Samples were annealed in a standard tube furnace with an Ar-2% H_2_ gas mixture. Milling and annealing procedures were the same as described in Reference 19.

### X-ray Diffraction

Powder x-ray diffraction was performed on both a Rigaku DMAX-Rapid Microdiffractometer equipped with a Mo x-ray source and a two-dimensional image plate and a Panalytical Empyrean^TM^ x-ray diffractometer with Cu Kα x-ray source and in the Bragg-Brentano geometry.

### Transmission Electron Microscopy

The microstructure of the samples was examined by transmission electron microscopy (TEM). Most TEM samples were prepared by focused ion-beam (FIB) milling to minimize the sample volume and thus magnetic interactions with the electron beam. (To preclude the possibility of FIB-induced artifacts from Ga implantation, control samples were also prepared by mechanical polishing the samples to 20–30 μm, followed by ion milling at low temperature to perforation. No significant differences between the differently prepared specimens were observed.) The TEM characterization was performed using a JEOL 2010F field-emission TEM operated at 200 kV and equipped with a scanning TEM (STEM) system, an energy dispersive x-ray spectroscopy (EDS) system (Oxford), and a Gatan Enfina electron energy loss (EEL) spectrometer. A JEOL 2000FX TEM microscope operated at 200 kV was used for large-angle tilting of the samples to obtain SAED patterns for multiple zone axes. Multislice simulations of diffraction pattern and HRTEM images for the proposed structure model were carried out using JEMS software^i^.

### First-Principles Calculations

All the first-principles calculations in the present work are performed using the projector augmented wave method (PAW)^42^,^43^ based Vienna ab-initio Simulation Package (VASP)^43^. Three different exchange-correlation functionals have been applied: (i) the general gradient approximation by Perdew, Burke, and Ernzerhof (PBE)^44^; (ii) the hybrid density functional Hartree-Fock (HF) method by Hey-Scuseria-Ernzerhof (HSE06), which is mixed by 25% of the exact HF exchange and 75% of the PBE exchange-correlation functional^45^,^46^,^47^; (iii) the hybrid functional HSEsol, which has the same form as HSE06, but is based on the PBEsol^48^ functional for the semi-local exchange and correlation part^49^. To increase the accuracy of the calculation, the plane wave energy cutoff is increased by a factor of 1.3 times the maximum energy of the pure elements. The structures are relaxed by implementing the Methfessel-Paxton method in order to calculate the forces acting on the atoms^50^. The atomic positions, volume, and cell shape of the proposed structures are relaxed with respect to all degrees of freedom by using high accuracy, spin-polarized calculations. The contour plots of the differential charge density were generated using VESTA^51^,^52^. The plots in Fig. 5D,E are the results achieved by PBE functional. In order to calculate the formation energy of the proposed Fe_3_O structure, BCC-Fe, HCP-Zr, and O_2_ molecule are also relaxed by VASP to obtain the ground state energy. The k-point grid was optimized for each calculation in order to achieve compromise between computing time and accuracy. For proposed Fe_3_O and (Fe,Zr)_3_O structure, a 20 × 20 × 20 grid is applied for the calculation based on PBE and a 9 × 9 × 9 grid is applied for both HSE06 and HSEsol. The k-point grids of BCC-Fe calculations are 20 × 20 × 20 for PBE and 11 × 11 × 11 for HSE06 and HSEsol. For HCP-Zr, 24 × 24 × 15 grid is chosen for PBE calculations and 11 × 11 × 6 for HSE06 and HSEsol. All the calculations of single oxygen atom and O_2_ molecule are relaxed by implementing Gamma centered scheme with k-meshes of 1 × 1 × 1.

## Additional Information

**How to cite this article**: Hu, Y.-J. *et al.* Nano-sized Superlattice Clusters Created by Oxygen Ordering in Mechanically Alloyed Fe Alloys. *Sci. Rep.* **5**, 11772; doi: 10.1038/srep11772 (2015).

## Supplementary Material

Supplementary Information

## References

[b1] Viswanathan R., Coleman K. & Rao U. Materials for ultra-supercritical coal-fired power plant boilers. Int. J. Pres. Ves. Pip. 83, 778–783 (2006).

[b2] Viswanathan R. *et al.* US program on materials technology for ultra-supercritical coal power plants. J. Mater. Eng. Perform. 14, 281–292 (2005).

[b3] Argon A. S. Strengthening mechanisms in crystal plasticity. (Oxford University Press: Oxford, , 2008).

[b4] Edwards G. A., Stiller K., Dunlop G. L. & Couper M. J. The precipitation sequence in Al-Mg-Si alloys. Acta Mater. 46, 3893–3904 (1998).

[b5] Reed R. C. The superalloys: fundamentals and applications. (Cambridge University Press, 2006).

[b6] Uchic M. D., Dimiduk D. M., Florando J. N. & Nix W. D. Sample dimensions influence strength and crystal plasticity. Science 305, 986–989 (2004). 15310897 10.1126/science.1098993

[b7] Zandbergen H. W., Andersen S. J. & Jansen J. Structure determination of Mg_5_Si_6_ particles in Al by dynamic electron diffraction studies. Science 277, 1221–1225 (1997).

[b8] Sato J. *et al.* Cobalt-base high-temperature alloys. Science 312, 90–91 (2006). 16601187 10.1126/science.1121738

[b9] Zener C. Theory of growth of spherical precipitates from solid solution. J. Appl. Phys. 20, 950–953 (2004).

[b10] Karabay S. Influence of AlB_2_ compound on elimination of incoherent precipitation in artificial aging of wires drawn from redraw rod extruded from billets cast of alloy AA-6101 by vertical direct chill casting. Mater. Des . 29, 1364–1375 (2008).

[b11] Monzen R., Iguchi M. & Jenkins M. Structural changes of 9R copper precipitates in an aged Fe-Cu alloy. Philos. Mag. Lett. 80, 137–148 (2000).

[b12] Calderon H., Fine M. & Weertman J. Coarsening and morphology of β′ particles in Fe-Ni-Al-Mo ferritic alloys. Metall. Trans. A 19, 1135–1146 (1988).

[b13] Stallybrass C. & Sauthoff G. Ferritic Fe–Al–Ni–Cr alloys with coherent precipitates for high-temperature applications. Mater. Sci. Eng., A 387, 985–990 (2004).

[b14] Koch C. C. Materials synthesis by mechanical alloying. Annu. Rev. Mater. Sci. 19, 121–143 (1989).

[b15] Suryanarayana C. Mechanical alloying and milling. Prog. Mater Sci. 46, 1–184 (2001).

[b16] Hirata A. *et al.* Atomic structure of nanoclusters in oxide-dispersion-strengthened steels. Nature Mater. 10, 922–926 (2011). 22019943 10.1038/nmat3150

[b17] Saber M. *et al.* Size effect of primary Y2O3 additions on the characteristics of the nanostructured ferritic ODS alloys: Comparing as-milled and as-milled/annealed alloys using S/TEM. J. Nucl. Mater. 452, 223–229 (2014).

[b18] Hirata A., Fujita T., Liu C. & Chen M. Characterization of oxide nanoprecipitates in an oxide dispersion strengthened 14YWT steel using aberration-corrected STEM. Acta Mater. 60, 5686–5696 (2012).

[b19] Darling K. A., VanLeeuwen B. K., Koch C. C. & Scattergood R. O. Thermal stability of nanocrystalline Fe-Zr alloys. Mater. Sci. Eng., A 527, 3572–3580 (2010).

[b20] Jang J. S. C. & Koch C. C. The hall-petch relationship in nanocrystalline iron produced by ball milling. Scripta Metall. Mater. 24, 1599–1604 (1990).

[b21] Belyakov A., Sakai Y., Hara T., Kimura Y. & Tsuzaki K. Effect of dispersed particles on microstructure evolved in iron under mechanical milling followed by consolidating rolling. Metall. Mat. Trans. A 32, 1769–1776 (2001).

[b22] Hidaka H., Kawasaki K., Tsuchiyama T. & Takaki S. Effect of carbon on nano-crystallization in steel during mechanical milling treatment. Mater. Trans., JIM 44, 1912–1918 (2003).

[b23] Khan A. S., Zhang H. & Takacs L. Mechanical response and modeling of fully compacted nanocrystalline iron and copper. Int. J. Plast. 16, 1459–1476 (2000).

[b24] Kotan H., Saber M., Koch C. & Scattergood R. Effect of annealing on microstructure, grain growth, and hardness of nanocrystalline Fe–Ni alloys prepared by mechanical alloying. Mater. Sci. Eng., A 552, 310–315 (2012).

[b25] Miller M. K., Russell K. F. & Hoelzer D. T. Characterization of precipitates in MA/ODS ferritic alloys. J. Nucl. Mater. 351, 261–268 (2006).

[b26] Moorthy V., Vaidyanathan S., Raj B., Jayakumar T. & Kashyap B. Insight into the microstructural characterization of ferritic steels using micromagnetic parameters. Metall. Mat. Trans. A 31, 1053–1065 (2000).

[b27] Mungole M., Sahoo G., Bhargava S. & Balasubramaniam R. Recrystalised grain morphology in 9Cr 1Mo ferritic steel. Mater. Sci. Eng., A 476, 140–145 (2008).

[b28] Qiu H., Ito R. & Hiraoka K. Role of grain size on the strength and ductile–brittle transition temperature in the dual-sized ferrite region of the heat-affected zone of ultra-fine grained steel. Mater. Sci. Eng., A 435, 648–652 (2006).

[b29] Kotan H., Darling K. A., Saber M., Scattergood R. O. & Koch C. C. Thermal stability and mechanical properties of nanocrystalline Fe–Ni–Zr alloys prepared by mechanical alloying. J. Mater. Sci. 48, 8402–8411 (2013).

[b30] Hirsch P. B., Howie A., Nicholson R., Pashley D. & Whelan M. J. Electron microscopy of thin crystals (Roberts E. Krieger Pulishing Company: New York, 1977).

[b31] Cullity B. D. & Stock S. R. Elements of X-ray diffraction. (Pearson, 2001).

[b32] Servant C., Gueneau C. & Ansara I. Experimental and thermodynamic assessment of the Fe-Zr system. J. Alloys Compd. 220, 19–26 (1995).

[b33] Li L. *et al.* High-temperature grain size stabilization of nanocrystalline Fe–Cr alloys with Hf additions. Mater. Sci. Eng., A 613, 289–295 (2014).

[b34] Burch T. J. *et al.* Hyperfine-field distribution in Fe_3_Si_1-x_Al_x_ alloys and a theoretical interpretation. Phys. Rev. B 19, 2933–2938 (1979).

[b35] Rademacher T., Al-Kassab T., Deges J. & Kirchheim R. Ordering and site occupancy of D0_3_ ordered Fe_3_Al-5 at%Cr evaluated by means of atom probe tomography. Ultramicroscopy 111, 719–724 (2011). 21247699 10.1016/j.ultramic.2010.12.009

[b36] Schönberg N. On the existence of a metallic molybdenum oxide. Acta Chem. Scad. 8, 617–619 (1954).

[b37] Gaffet E., Louison C., Harmelin M. & Faudot F. Metastable phase transformations induced by ball-milling in the Cu-W system. Mater. Sci. Eng., A 134, 1380–1384 (1991).

[b38] Miller M. K., Hoelzer D. T., Kenik E. A. & Russell K. F. Nanometer scale precipitation in ferritic MA/ODS alloy MA957. J. Nucl. Mater. 329, 338–341 (2004).

[b39] Ratti M., Leuvrey D., Mathon M. H. & de Carlan Y. Influence of titanium on nano-cluster (Y, Ti, O) stability in ODS ferritic materials. J. Nucl. Mater. 386-88, 540–543 (2009).

[b40] Xu J., Liu C. T., Miller M. K. & Chen H. M. Nanocluster-associated vacancies in nanocluster-strengthened ferritic steel as seen via positron-lifetime spectroscopy. Phys. Rev. B 79 (2009).

[b41] Xu W. Z. *et al.* Nano ZrO_2_ particles in nanocrystalline Fe–14Cr–1.5Zr alloy powders. J. Nucl. Mater. 452, 434–439 (2014).

[b42] Bloechl P. E. Projector augmented-wave method. Phys. Rev. B 50, 17953 (1994). 10.1103/physrevb.50.17953 9976227

[b43] Kresse G. & Furthmueller J. Efficient iterative schemes for ab initio total-energy calculations using a plane-wave basis set. Phys. Rev. B 54, 11169 (1996). 10.1103/physrevb.54.11169 9984901

[b44] Perdew J. P., Burke K. & Ernzerhof M. Generalized gradient approximation made simple. Phys. Rev. Lett. 77, 3865–3868 (1996). 10062328 10.1103/PhysRevLett.77.3865

[b45] Heyd J., Scuseria G. E. & Ernzerhof M. Hybrid functionals based on a screened Coulomb potential. J. Chem. Phys. 118, 8207–8215 (2003).

[b46] Heyd J., Scuseria G. E. & Ernzerhof M. Erratum: “Hybrid functionals based on a screened Coulomb potential” [J. Chem. Phys.118, 8207 (2003)]. J. Chem. Phys. 124, 219906 (2006).

[b47] Paier J. *et al.* Erratum: “Screened hybrid density functionals applied to solids” [J. Chem. Phys.124, 154709 (2006)]. J. Chem. Phys. 125, 249901 (2006). 10.1063/1.2187006 16674253

[b48] Perdew J. P. *et al.* Restoring the Density-Gradient Expansion for Exchange in Solids and Surfaces. Phys. Rev. Lett. 100, 136406 (2008). 18517979 10.1103/PhysRevLett.100.136406

[b49] Schimka L., Harl J. & Kresse G. Improved hybrid functional for solids: The HSEsol functional. J. Chem. Phys. 134, 024116 (2011). 21241089 10.1063/1.3524336

[b50] Methfessel M. & Paxton A. T. High-Precision Sampling for Brillouin-Zone Integration in Metals. Phys. Rev. B 40, 3616–3621 (1989). 10.1103/physrevb.40.3616 9992329

[b51] Momma K. & Izumi F. VESTA: a three-dimensional visualization system for electronic and structural analysis. J. Appl. Crystallogr. 41, 653–658 (2008).

[b52] Momma K. & Izumi F. VESTA 3 for three-dimensional visualization of crystal, volumetric and morphology data. J. Appl. Crystallogr. 44, 1272–1276 (2011).

