# Supplementary material for: Nano-sized Superlattice Clusters Created by Oxygen Ordering in Mechanically Alloyed Fe Alloys
Source: Sci Rep. 2015 Jul 2;5:11772. doi: 10.1038/srep11772 (PMC4650680; doi:10.1038/srep11772)
Supplement: Supplementary Information [file srep11772-s1.doc]

**SUPPLEMENTARY INFORMATION**

**Nano-sized Superlattice Clusters Created by Oxygen Ordering in Mechanically Alloyed Fe Alloys**

Yong-Jie Hu^1^, Jing Li^2^, Kristopher A. Darling^3^, William Y. Wang^1^, Brian K. VanLeeuwen^1^, Xuan Liu^1^, Laszlo J. Kecskes^3^, Elizabeth C. Dickey^2^, and Zi-Kui Liu^1^

^1^ Department of Materials Science and Engineering, The Pennsylvania State University, University Park, PA 16802, USA

^2^ Department of Materials Science and Engineering, North Carolina State University, Raleigh, NC 27695, USA

^3^ U.S. Army Research Laboratory, Weapons and Materials Research Directorate, RDRL-WMM-F, Aberdeen Proving Ground, MD 21005, USA

**Supplementary text**

**1. X-ray diffraction data**

The X-ray diffraction (XRD) pattern of the ball-milled α-Fe sample (unalloyed Fe) annealed at 913 °C for one hour shows only the presence of α-Fe (Figure S1A), while that of the similarly prepared 1at% Zr sample shows minor traces of Fe_3_Zr (Figure S1B). In neither sample are Fe-oxide phases detected by XRD.


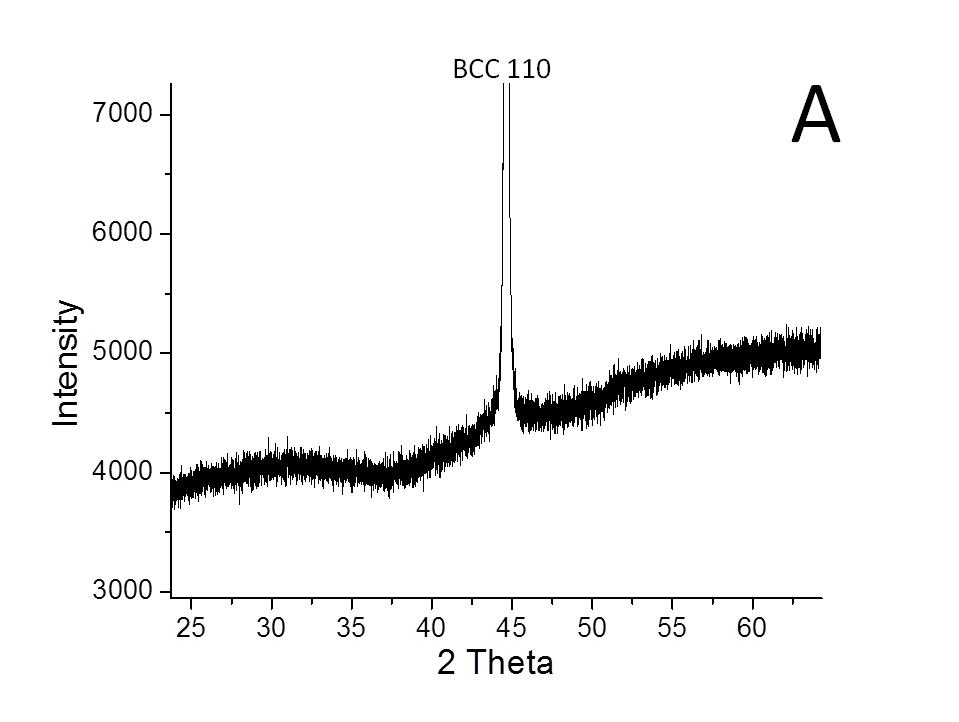


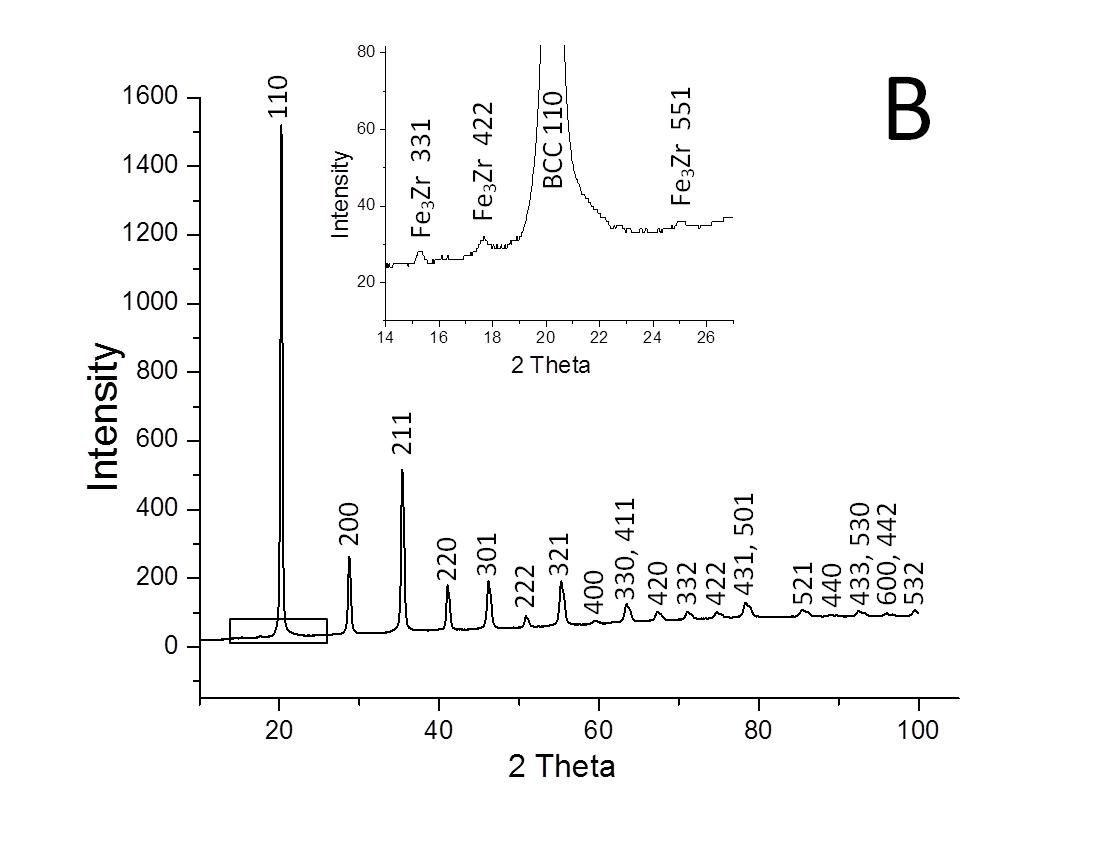


**Figure S1.** (A) XRD data (Cu Kα radiation) for the unalloyed α-Fe sample near the α-Fe (110) reflection. No secondary phase peaks were observed in this sample. (B) XRD data (Mo Kα radiation) for the Fe-1at% Zr sample. The inset shows enlarged data around the α-Fe (110) peak (marked with box); the three small extra peaks correspond closely to Fe_3_Zr.

**2. Chemical compositions of as-milled powder and annealed sample**

The chemical compositions of as-milled Fe-1at%Zr powders is carefully determined, in terms of different elements (Table S1). As shown in Table S1, the powders are mainly composed of Fe, and have about 0.98wt% O and 1.48wt% Zr. The contents of other possible oxide-former elements in ODS, such as Ti, Y, Hf and Al, are almost zero. In addition, the contents of elements C, S and Si are also very low.

**Table S1.** Chemical compositions of as milled Fe-1at%Zr powders (wt.%)

| O | N | C | S | Si | Cr | Ni |
| --- | --- | --- | --- | --- | --- | --- |
| 0.989 | 0.025 | 0.024 | 0.0008 | 0.048 | 0.30 | 0.039 |
| Zr | Y | Ti | La | Al | Hf | Fe |
| 1.43 | <0.0005 | 0.017 | <0.002 | 0.048 | <0.002 | Balance |

Figure S2A shows a TEM bright field (BF) image of α-Fe matrix in the Fe-1at% Zr sample. In some local areas, some secondary phase particles with low density and size ranging 15-30 nm (a few 50 nm) are observable. Full EDS spectra analysis indicated that for the α-Fe matrix of the ball-milled Fe-1at.% Zr sample there is no additional element except Fe and Zr (O element was not considered because of TEM sample surface oxidation). The Zr concentration is very low for the α-Fe matrix (see Figure S2B, and the tiny Ga peak is due to the beam source of FIB), while the secondary phase particles are Zr enriched (Figure S2C), which is in agreement with XRD result (Fe_3_Zr particle).


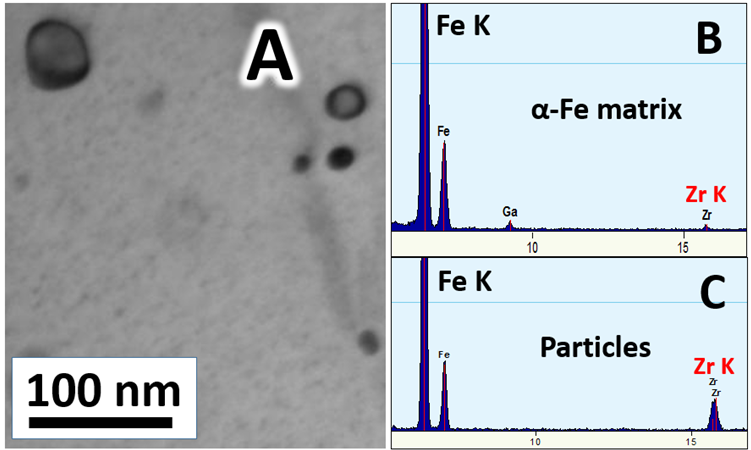


**Figure S2.** (A) TEM micrograph of the α-Fe matrix of the ball-milled Fe-1at% Zr sample showing some secondary phase particles with low density and size ranging 15-30 nm (a few 50 nm). (B) EDS spectrum of the α-Fe matrix in (A) showing Fe and very low Zr (tiny Ga peak came from FIB sample preparation). (C) EDS spectrum of the particles in (A), showing that the particles are significantly Zr enriched.

**3. TEM characterization of the nano-sized superlattice (NSS) clusters in the Fe-1at.% Zr samples**

The morphology, size, and distribution of the superlattice phase in the Fe-1at.% Zr sample have been identified using TEM dark-field imaging, showing dispersed NSS clusters with a size ranging 2-5.5 nm, similar to that of the unalloyed Fe sample, see Figure S3A (SAED) and Figure S3B (DF image).


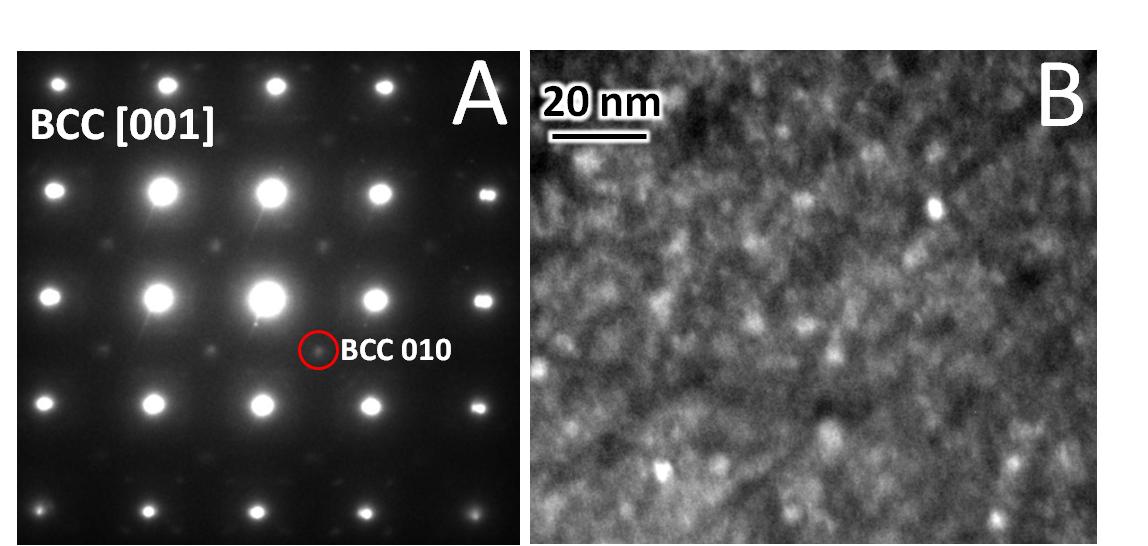


**Figure S3.** (A) SAED pattern of the Fe-1at.% Zr sample oriented along α-Fe [001] showing {100} superlattice reflections. (B) Dark-field image using the (010) superlattice reflection in (A) showing dispersed phase with a size ranging 2-5.5 nm.

**4. TEM analysis for surface Spinel Fe oxide by EELS and SAED**

In order to examine if there is surface oxidation, EELS analysis was carried out on the unalloyed Fe and Fe-1at.%Zr TEM thin samples with gradually changed sample thickness along electron beam direction. The sample thickness, T, for different areas, was identified by the T/λ (λ: mean free path of inelastic scattering) which can be measured on low loss EELS spectrum recorded at corresponding area. The oxygen concentration for these different areas was provided by the Gatan “Digital Micrograph” EELS quantification software. It was clearly shown that the intensity of the oxygen K-edge reduced with increasing sample thickness (Figures S4A and S4B). Figure S4C shows the plots of oxygen concentration versus sample thickness, indicating that the oxygen concentration decreases with the gradually increased sample thickness. This is indicative of surface oxidation, which may be produced when the TEM samples were exposed to air before the TEM examinations.


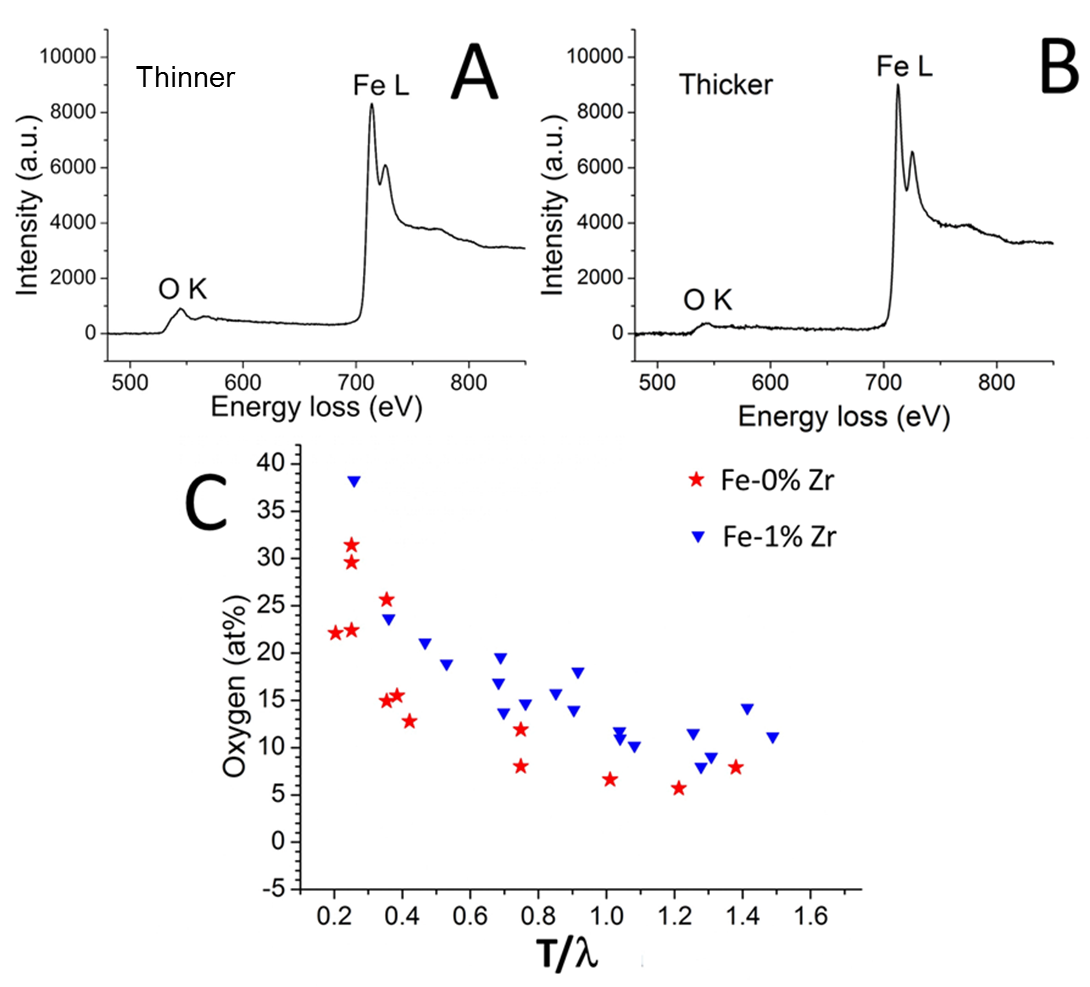


Figure S4. EELS spectra for thinner (A) and thicker (B) TEM sample areas showing different oxygen K-edge intensities. (C) Plots for oxygen concentration vs. TEM sample thickness. The sample thickness, T, was identified by the value of T/λ with λ being the mean free path of inelastic scattering. The oxygen concentration decreases with increasing TEM sample thickness, indicative of the existence of surface oxides.

The structure of the surface oxide was further characterized using SAED. Figure S5A shows a α-Fe grain initially oriented along
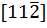
 in the Fe-1at.% Zr sample. There is an additional pattern which can be identified as spinel Fe_3_O_4_ oriented along
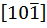
. The orientation relationship between α-Fe and Fe_3_O_4_ for this particular grain is:_α-Fe_//_Fe3O4_ and _α-Fe_//(040)_Fe3O4_; same as that reported in the literature[^1^](#_ENREF_1). Figures S5B and S5C show SAED patterns obtained by tilting this α-Fe grain along
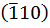
* to
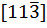
 (Figure S5B) and
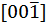
 (Figure S5C), respectively. All patterns show additional reflections from the surface Fe_3_O_4_ or γ-Fe_2_O_3_ [note, the lattice parameter a_0_ = 8.31 Å is very similar to that of Fe_3_O_4_ (a_0_= 8.40 Å)].


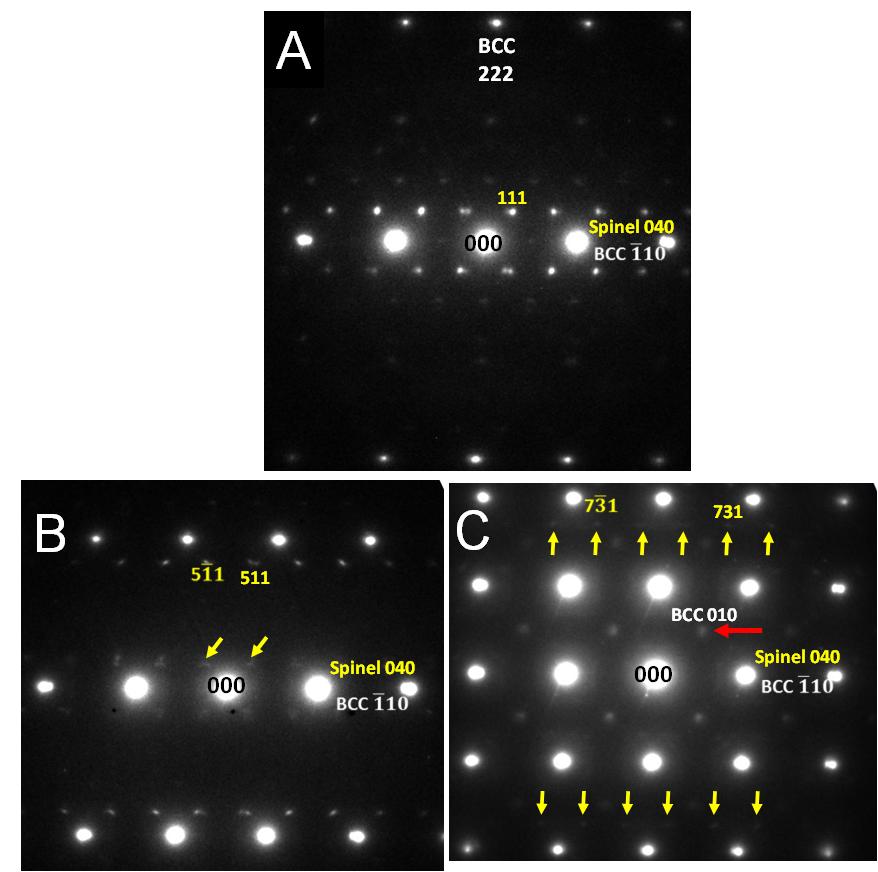


**Figure S5.** (A) α-Fe //Fe_3_O_4_; (B) α-Fe //γ-Fe_2_O_3_ and (C) α-Fe //Fe_3_O_4_. In (B) the inclined arrows mark double diffraction spots; in (C) the vertical arrows mark Fe_3_O_4_, while the horizontal, red arrow marks a set of reflections not due to Fe_3_O_4_ but instead which correspond to α-Fe {100} forbidden reflections. (sample: Fe-1at.% Zr)

**5. Confirmation of α-Fe {100} forbidden reflections in bulk Fe matrix**

In Figure S5C (Fe-1at.% Zr sample), when α-Fe was tilted along
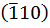
* from an initial (Figure S5A) to
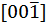
, the Fe_3_O_4_ was correspondingly tilted to
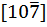
 (α-Fe
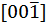
//Fe_3_O_4_
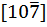
). It is clearly shown in Figure S4C that there is an additional set of reflections not due to Fe_3_O_4_ or γ-Fe_2_O_3_ (marked by the red arrow), but instead which correspond to α-Fe {100} forbidden reflections. Similarly, in Figure S6A for an α-Fe grain in the Fe sample, the original orientation relationship is close to [115]_α-Fe_ //[001]_Fe3O4_; after tilting α-Fe along
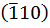
* from [115] to [001], the α-Fe {100} forbidden reflections show up again (see Figure 2A, the same α-Fe grain shown as in Figure S6A).


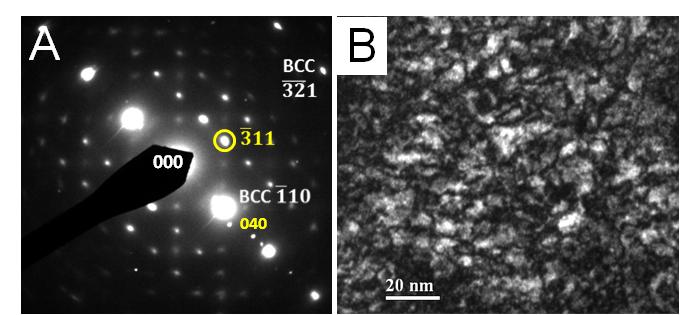


**Figure S6.** SAED pattern and dark-field image of surface spinel Fe**_3_**O**_4_** in the Fe sample. (A) SAED pattern for close to α-Fe [115]//Fe_3_O_4_ [001]. The reflections from [001] first order Laue zone of Fe_3_O_4_ are evident in the diffraction pattern. (B) dark-field image using a (
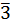
11) spinel Fe_3_O_4_ reflection marked in (A), showing morphology (irregular) and size (10-20 nm) of the surface spinel Fe_3_O_4_.

Many orientation relationships between the α-Fe and surface spinel Fe oxide have been observed in the unalloyed Fe and Fe-1at.% Zr samples (e.g., Figures S5A and S6A), which is in agreement with the literature that the orientation relationship depends on the sample surface normal[^1^](#_ENREF_1). But, when the α-Fe grains were tilted into <100> axes, the α-Fe {100} forbidden reflections consistently showed up regardless the initial orientation relationship. This indicates that the α-Fe {100} forbidden reflections are a bulk phenomenon, are not caused by the surface spinel oxide(s), and are due to ordering in the bulk α-Fe matrix (supperlattice). Also, the surface spinel Fe_3_O_4_ has its own morphology (irregular) and has its own size range (10-20 nm) which are significantly different from those of the NSS clusters, see Figure S6B.

**6. TEM SAED analysis of the starting Fe powders**

The starting Fe powders (before ball-milling) were examined by TEM to demonstrate the existence of polycrystal spinel Fe_3_O_4_, see Figure S7. No {100} superlattice reflections are present. It is likely that the pre-existing surface oxides on the initial powders are dissolved into α-Fe matrix during high-energy ball milling and provide the source of oxygen to form NSS clusters. A similar phenomenon has been reported by Korznikov et al.[^2^](#_ENREF_2), in which highly supersaturated levels of oxygen in α-Fe are achieved by large plastic deformation via shear compaction of nano-scale Fe powders containing ~15 vol% of surface oxides. In that study, FeO particles with an average size of 200 nm were found to precipitate at annealing temperatures of 600 °C; this is in contrast to our findings in which secondary oxide phases are not observed even up to annealing temperatures of 913 °C, with oxygen remaining incorporated and ordered in the α-Fe lattice.


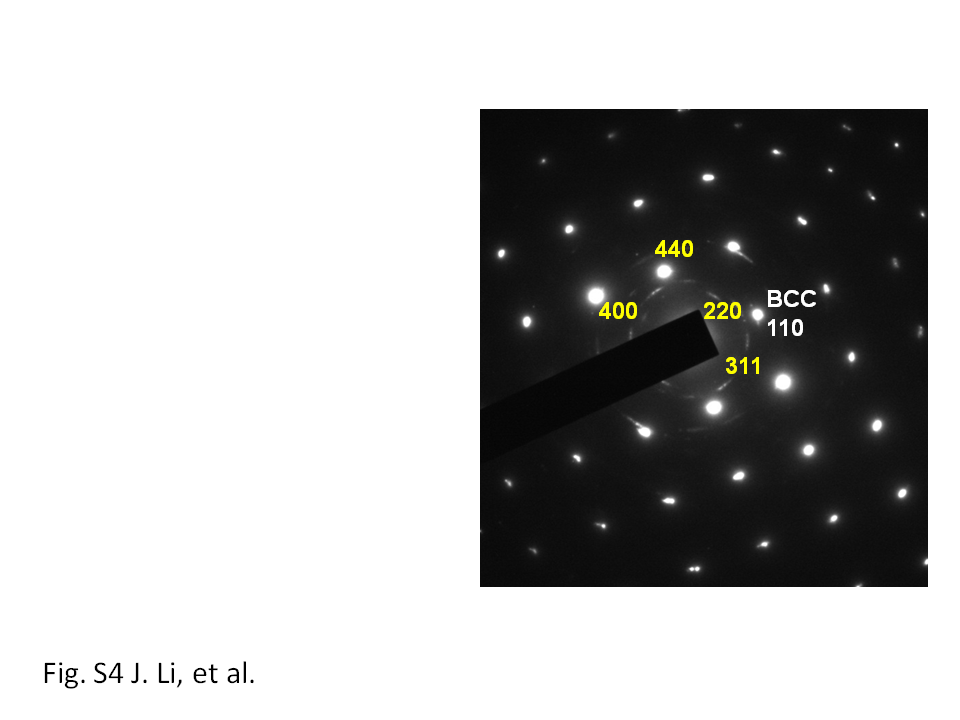


**Figure S7.** [001] SAED pattern of a BCC Fe grain in the starting Fe powder showing polycrystal reflections from the spinel Fe_3_O_4_ on the TEM sample surface, but there are no superlattice reflections (compare with Figure 2A).

**7. SAED simulation of the proposed Fe_3_O structure**

The electron diffraction patterns of Fe_3_O and α-Fe structures along different zone axes were simulated by using a JEMS software to compare them with each other and with those observed experimentally, and to verify the proposed Fe_3_O structure model. See Figure S8.


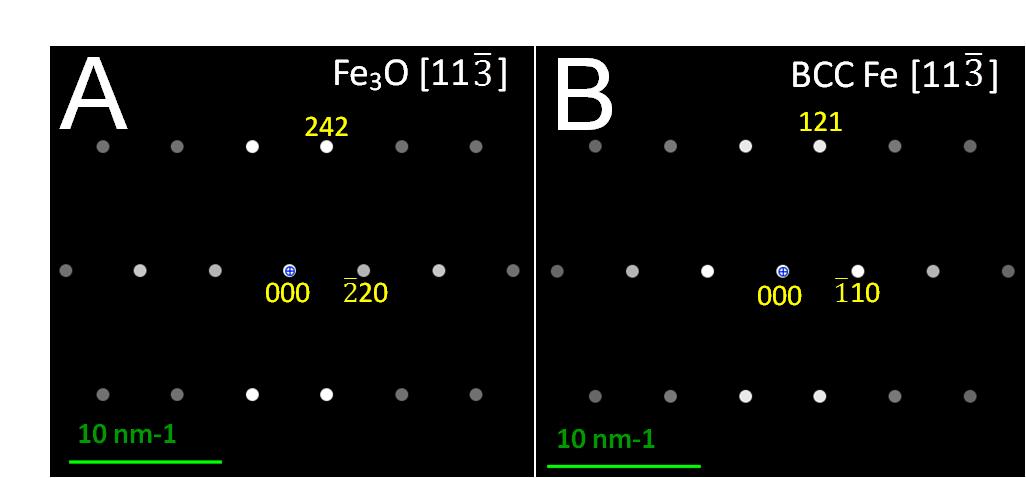


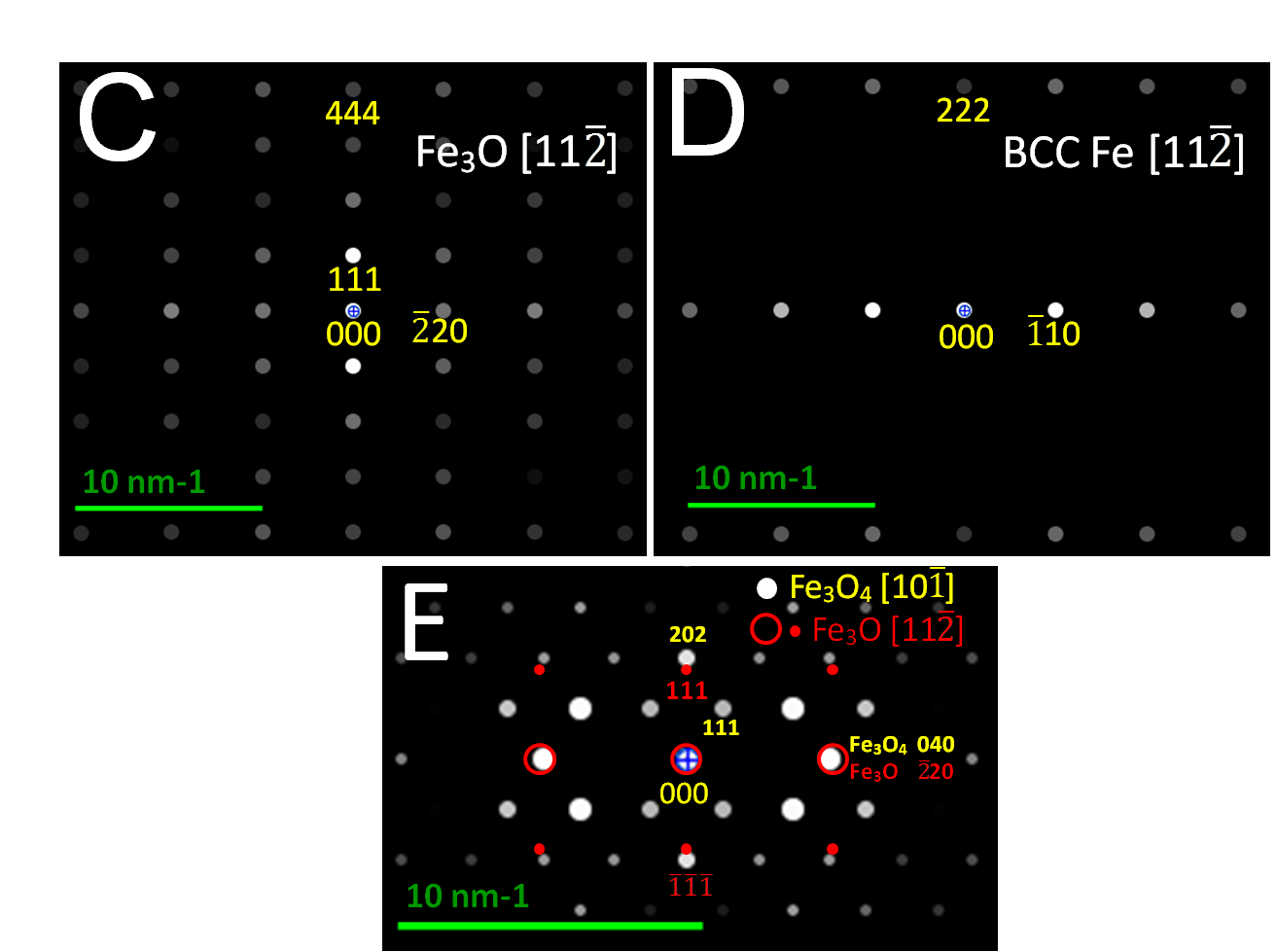


Figure S8. Simulated SAED patterns for the proposed Fe_3_O structure along different zone axes compared with those of α-Fe. (A) Simulated SAED patterns for Fe_3_O along the [11
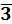
] zone axis. (B) Simulated BCC α-Fe pattern along the [11
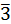
] zone axis. (C) Simulated Fe_3_O pattern along the [11
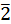
]. (D) Simulated α-Fe pattern along the [11
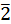
] zone axis. (E) Simulated SAED pattern for surface Fe_3_O_4_ along the [10
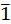
] zone axis. The red circles and dots mark the positions of the [11
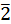
] reflections of Fe_3_O [copied from (C)].

Comparing (A) with (B) indicates that the Fe_3_O structure will not create additional reflection on the [11
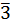
] α-Fe SAED pattern. This is in good agreement with experimental observations (See Figure S5B). Comparing (C) with (D), it is shown that the Fe_3_O structure creates extra superlattice reflections on the α-Fe [11
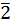
] SAED pattern. However, as seen from (E), the diffraction pattern of Fe_3_O along [11
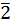
] direction are either overlap (red circles) or are very close (red dots) to those of the surface Fe_3_O_4_. It is reasonable to assume that due to the existence of the [10
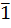
] SAED pattern of surface Fe_3_O_4_ along this zone axis, the extra superlattice reflections of [11
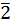
] Fe_3_O may be very dim and could not be observed in the pattern for α-Fe // Fe_3_O_4_ (See Figure S5A)

**8. First-principles calculations**

As shown in previous calculations, the two common DFT electron exchange-correlation functionals, the local-density (LDA) and the generalized gradient approximation (GGA), lead to considerable errors in calculated redox energies, especially for 3d transition metals oxides . The errors can be attributed to self-interaction error in electron density during electrons transfer and localization[^3^](#_ENREF_3). It is found such errors can be corrected by using the DFT+U approach and modifying the ground state energy of O_2_ molecule based on experimental binding energy[^3^](#_ENREF_3). The empirically determined U parameter is chosen by comparing with various experimental data, which means such method can only be applied to oxides which have available experimental measurements.

The hybrid exchange-correlation functional, HSE06, has been shown to reproduce accurately redox energies of transition metal oxides. In the hybrid approach, Hartree-Fock exchange is taken into account to partially cancel the electron self-interaction error mentioned above. In addition, HSE06, unlike the U parameter approach, is a parameter free functional that can accurately study oxides binding for which no experimental measurements exit. Unfortunately, HSE06 was found to have tendency to overestimate lattice constants, which is pronounced in various metal elements, including Fe[^8-10^](#_ENREF_8). Additionally, the atomization energies of transition metals exhibit significantly increased errors compared to PBE[^11^](#_ENREF_11). To further correct the errors of lattice parameter and atomization energies in the HSE06 functional, another hybrid functional, HSEsol, was introduced recently[^11^](#_ENREF_11). The HSEsol functional has the same form as HSE06, but use the PBEsol functional for the semi-local exchange and correlation part instead of PBE[^11^](#_ENREF_11). Compared to HSE06, HSEsol exhibits better predictions of lattice parameters for some transition metals (Cu, Pd, Ag and Rh) [^11^](#_ENREF_11). However, it has not been demonstrated that HSEsol can also reproduce redox energies of transition metals as well as HSE06. Therefore, in order to study the proposed Fe_3_O structure comprehensively and systematically, these three functionals, PBE, HSE06 and HSEsol, are all employed in the present work.

The equilibrium state of the pure metal elements and compounds in the present work,
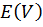
, is determined by fitting the total energy vs. volume based on the four-parameter Birch-Murnaghan equation of state (EOS)[^12^](#_ENREF_12)


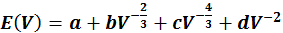
 (1)

where a, b, c, and d are fitting parameters, from which the equilibrium volume (V_0_) and ground state energy can be obtained. In order to validate the calculations of redox energies, α-Fe_2_O_3_ is also included.

The lattice parameters of BCC-Fe, HCP-Zr, α-Fe_2_O_3_ , Fe_3_O and (Fe,Zr)_3_O calculated from different exchange-correlation functionals are summarized in Table S1. For (Fe,Zr)_3_O, the atomic positions of Zr are considered for two possible cases due to the symmetry: Zr occupies the first nearest neighbor site (FNS) or the second nearest neighbor site (SNS) with respect to the oxygen atom. Experimental data and other first-principles calculations results are also included in Table S1 for comparison. In addition, for BCC-Fe, HCP-Zr and α-Fe_2_O_3_, we report the corresponding relative error of calculated results with respect to the experimental values. Table S1 reveals that both PBE and HSEsol provide good predictions of the lattice parameters of BCC-Fe, HCP-Zr and α-Fe_2_O_3_, giving errors within 1% compared to experimental data. The prediction of HCP-Zr and α-Fe_2_O_3_ achieved by HSEsol is also in good agreement with experiments. However, for BCC-Fe, HSEsol significantly overestimates the lattice parameter, which is 2.0% larger than experimental values. Similar result was reported in other calculations as well [^8^](#_ENREF_8).

In order to investigate the coherency between the proposed Fe_3_O structure and the BCC-Fe matrix, we calculate the lattice mismatch of Fe_3_O with respect to both experimental and calculated lattice parameters of BCC-Fe. The results are listed in Table S2. As seen from the table, very small lattice mismatch is achieved by all three functionals, with respect to experimental lattice parameter of BCC-Fe. The average lattice mismatch is about -0.7%, which indicates a good coherency between the Fe_3_O and BCC-Fe, in agreement with the HRTEM observation. The values of lattice mismatch with respect to the calculated lattice parameter are very different. For the PBE functional, the lattice mismatch is as small as 0.3%, while HSE06 gives a large mismatch of -2.1%, which could be attributed to the bad estimation on the lattice parameter of BCC-Fe.

**Table S2.** Lattice parameters of BCC-Fe, HCP-Zr, α-Fe_2_O_3_ , Fe_3_O and (Fe,Zr)_3_O calculated by the PBE, HSE06 and HSEsol functionals. The other first-principles calculations results by using same functionals are also included. The unit of lattice parameters is Å. For BCC-Fe, HCP-Zr and α-Fe_2_O_3_, the comparison with experimental lattice parameters is given in terms of the relative error. The lattice mismatch is calculated as the relative difference between the lattice parameter of Fe_3_O and the two times of the BCC-Fe lattice parameter.

|  | **Fe** | **Zr** | | **α-Fe_2_O_3_** | | **Fe_3_O** | **(Fe,Zr)_3_O** | |
| --- | --- | --- | --- | --- | --- | --- | --- | --- |
|  |  | a | c | a | c |  | SNS | FNS |
| **Expt.** | 2.861^a^ | 3.230^b^ | 5.141^b^ | 5.035^c^ | 13.747 ^c^ |  |  |  |
| **PBE** | 2.832 | 3.233 | 5.147 | 5.045 | 13.801 | 5.679 | 5.755 | 5.859 |
|  | 2.832^d^ | 3.240^e^ | 5.178 ^e^ | 5.016^f^ | 13.876 ^f^ |  |  |  |
|  | -1.0% | +0.1% | +0.1% | +0.2% | +0.4% | -0.7%  +0.3% |  |  |
| **HSE06** | 2.919 | 3.255 | 5.160 | 5.035 | 13.723 | 5.714 | 5.912 | 6.104 |
|  | 2.909^d^ |  |  | 5.035 ^f^ | 13.763 ^f^ |  |  |  |
|  | +2.0% | +0.6% | +0.3% | 0.0% | -0.2% | -0.1%  -2.1% |  |  |
| **HSEsol** | 2.872 | 3.218 | 5.093 | 5.009 | 13.652 | 5.646 | 5.761 | 5.930 |
|  | +0.4% | -0.4% | -0.9% | -0.5% | -0.7% | -1.3%  -1.7% |  |  |

a Experimental measured lattice parameter of BCC-Fe at 93K[^13^](#_ENREF_13)

b Experimental measured lattice parameter of HCP-Fe at 4.2K[^14^](#_ENREF_14)

c Experimental measured lattice parameter of α-Fe_2_O_3_ at 273K[^15^](#_ENREF_15)

d Calculated lattice parameter of BCC-Fe by other first-principles calculations

e Calculated lattice parameter of HCP-Zr by other first-principles calculation[^17^](#_ENREF_17)

f Calculated lattice parameter of α-Fe_2_O_3_ by other first-principles calculation[^18^](#_ENREF_18)

Table S3 reports the ground state energies (E_0_) of a single O atom and the O_2_ molecule calculated by using PBE, HSE06 and HSEsol functionals. Based on the ground state energy, the binding energy (ΔE) of the O_2_ molecule is calculated and compared with the experimental value. As seen from the table, PBE predicts a binding energy of -6.04eV, which is considerably higher than the experimental value. Similar results were also reported in the literatures. HSEsol also overestimates the binding energy of O_2_ molecule by 0.44eV. In contrast, the HSE06 calculation yields a binding energy of -5.15 eV, in much better agreement with experiments compared to the predictions of PBE and HSEsol. In order to eliminate the effects of such overestimations on the further calculations of formation enthalpies, we also give the corrected ground state energy of O_2_ molecule which is modified based on the experimental binding energy.

**Table S3.** The ground state energies (E_0_) of single O atom and O_2_ molecule, and the binding energy (ΔE) of O_2_ molecule calculated by using PBE, HSE06 and HSEsol functionals. The differences between the calculated binding energies and experimental data are listed below the ΔE. The corrected ground state energy of the O_2_ molecule is calculated via E_0_^corrected^ = 2E_0_( O atom)+ ΔE^Expt.^. The unit of all the data in Table S2 is eV.

|  | **PBE** | **HSE06** | **HSEsol** | **Expt.** |
| --- | --- | --- | --- | --- |
| **E_0_ (O atom)** | -1.91 | -5.94 | -5.46 |  |
| **E_0_ (O_2_ molecule)** | -9.86 | -17.04 | -16.48 |  |
| **ΔE** | -6.04  +0.91 | -5.15  +0.02 | -5.57  +0.44 | -5.13^a^ |
| **E_0_^corrected^(O_2_ molecule)** | -8.95 | -17.02 | -16.04 |  |

a Reference [^20^](#_ENREF_20)

Two types of 0K formation enthalpies of α-Fe_2_O_3_, the proposed Fe_3_O and (Fe,Zr)_3_O structures with respect to BCC-Fe, HCP-Zr and the O_2_ molecule are listed in Table S4. The ΔH is calculated by using the ground state energy of O_2_ molecule from direct DFT calculations and the ΔH^corrected^ is based on the corrected ground state energies of the O_2_ molecule shown in Table S2. As seen from Table S3, PBE significantly underestimates the formation enthalpies of α-Fe_2_O_3_ which confirms the previous argument in the beginning of this chapter. Even with the ground state correction for the O_2_ molecule, the formation enthalpy is still 18.3 kJ/mol-atom lower than the experimental value. In contrast, the HSE06 functional provides much better prediction of the formation enthalpy of α-Fe_2_O_3_ as expected. Besides, the HSEsol functional overestimates the formation enthalpies of α-Fe_2_O_3_ for both corrected and uncorrected cases. Therefore, it can be concluded that the HSE06 functional would provide more reliable formation enthalpies of the proposed Fe_3_O and (Fe,Zr)_3_O structures rather than the other two functionals.

In addition, it is revealed that the formation enthalpy of (Fe,Zr)_3_O (FNS) structure is much lower than that of the (Fe,Zr)_3_O (SNS) structure, which means that the former structure is more stable at equilibrium state. However, given that the FNS structure has larger lattice parameter than the SNS structure while constrained in BCC-Fe matrix, it is important to compare their energies under constrained conditions. Constrained state energies could be achieved via the E-V fittings which provides the volume dependence of the total energy. It is found that the constrained state energy of the FNS structure is still much lower than it of the SNS structure. Therefore, it is proposed that the Zr will prefer to occupy the nearest neighbor rather than second nearest neighbor sites of oxygen.

**Table S4.** 0K formation enthalpies of α-Fe_2_O_3_, the proposed Fe_3_O and (Fe,Zr)_3_O structures with respect to BCC-Fe, HCP-Zr and O_2_ molecule. The ΔH is calculated by using the ground state energy of O_2_ molecule from direct DFT calculations and the ΔH^corrected^ is calculated based on the corrected ground state energies of O_2_ molecule in Table S2. The unit of all the formation enthalpies is kJ/mol-atom.

|  | **Fe_2_O_3_** | | **Fe_3_O** | | **(Fe,Zr)_3_O (SNS)** | | **(Fe,Zr)_3_O (FNS)** | |
| --- | --- | --- | --- | --- | --- | --- | --- | --- |
|  | **ΔH** | **ΔH^corrected^** | **ΔH** | **ΔH^corrected^** | **ΔH** | **ΔH^corrected^** | **ΔH** | **ΔH^corrected^** |
| **Expt.** | -163.8^a^ |  |  |  |  |  |  |  |
| **PBE** | -119.2 | -145.5 | 40.4 | 29.4 | 37.9 | 27.1 | 17.9 | 7.6 |
| **HSE06** | -173.6 | -174.2 | 39.0 | 38.4 | 42.7 | 42.1 | 12.6 | 12.0 |
| **HSEsol** | -177.9 | -190.7 | 38.7 | 33.5 | 43.6 | 38.4 | 17.1 | 11.8 |

a Reference [^20^](#_ENREF_20)

**References and note**

1 Foley, C. L., Kruger, J. & Bechtold., C.J., Electron Diffraction Studies of Active Passive and Transpassive Oxide Films Formed on Iron. *Journal of the Electrochemical Society* **114**, 994-1001 (1967).

2 Korznikov, A. V., Safarov, I. M., Laptionok, D. V. & Valiev, R. Z. Structure and properties of superfine-grained iron compacted out of ultradisperse powder. *Acta Metall Mater* **39**, 3193-3197 (1991).

3 Wang, L., Maxisch, T. & Ceder, G. Oxidation energies of transition metal oxides within the GGA+ U framework. *Physical Review B* **73**, 195107 (2006).

4 Zhou, F., Cococcioni, M., Marianetti, C. A., Morgan, D. & Ceder, G. First-principles prediction of redox potentials in transition-metal compounds with LDA+U. *Physical Review B* **70**, 235121 (2004).

5 Jomard, G., Amadon, B., Bottin, F. & Torrent, M. Structural, thermodynamic, and electronic properties of plutonium oxides from first principles. *Physical Review B* **78**, 075125 (2008).

6 Chevrier, V. L., Ong, S. P., Armiento, R., Chan, M. K. Y. & Ceder, G. Hybrid density functional calculations of redox potentials and formation energies of transition metal compounds. *Physical Review B* **82**, 075122 (2010).

7 Franchini, C., Podloucky, R., Paier, J., Marsman, M. & Kresse, G. Ground-state properties of multivalent manganese oxides: Density functional and hybrid density functional calculations. *Physical Review B* **75**, 195128 (2007).

8 Jang, Y.R. & Yu, B. D. Structural, Magnetic, and Electronic Properties of Fe: A Screened Hybrid Functional Study. *Journal of Magnetics* **16**, 201-205 (2011).

9 Krukau, A. V., Vydrov, O. A., Izmaylov, A. F. & Scuseria, G. E. Influence of the exchange screening parameter on the performance of screened hybrid functionals. *The Journal of Chemical Physics* **125**, 224106 (2006).

10 Paier, J. *et al.* Screened hybrid density functionals applied to solids. *The Journal of Chemical Physics* **124**, 154709 (2006).

11 Schimka, L., Harl, J. & Kresse, G. Improved hybrid functional for solids: The HSEsol functional. *Journal of Chemical Physics* **134**, 024116 (2011).

12 Shang, S. L., Wang, Y., Kim, D. & Liu, Z. K. First-principles thermodynamics from phonon and Debye model: Application to Ni and Ni3Al. *Computational Materials Science* **47**, 1040-1048 (2010).

13 Villars, P. & Calvert, L. D. *Pearson's handbook of crystallographic data for intermetallic phases*. Vol. 2 (American Society for Metals Metals Park, OH, 1985).

14 Goldak, J., Lloyd, L. T. & Barrett, C. S. Lattice Parameters, Thermal expansions, and grüneisen coefficients of zirconium, 4.2 to 1130 K. *Physical Review* **144**, 478-484 (1966).

15 Olsen, J. S., Cousins, C., Gerward, L., Jhans, H. t. & Sheldon, B. A study of the crystal structure of Fe_2_O_3_ in the pressure range up to 65 GPa using synchrotron radiation. *Physica Scripta* **43**, 327 (1991).

16 Shang, S. L. *et al.* Vacancy mechanism of oxygen diffusivity in bcc Fe: A first-principles study. *Corrosion Science* **83**, 94-102 (2014).

17 Hao, Y.J., Zhang, L., Chen, X.R., Li, Y.H. & He, H.L. Phase transition and elastic constants of zirconium from first-principles calculations. *Journal of Physics: Condensed Matter* **20**, 235230 (2008).

18 Liao, P. & Carter, E. A. Testing variations of the GW approximation on strongly correlated transition metal oxides: hematite (α -Fe2O3) as a benchmark. *Physical Chemistry Chemical Physics* **13**, 15189-15199 (2011).

19 Hammer, B., Hansen, L. B. & Nørskov, J. K. Improved adsorption energetics within density-functional theory using revised Perdew-Burke-Ernzerhof functionals. *Physical Review B* **59**, 7413-7421 (1999).

20 Chase, M. W. & Force, J. A. N. A. NIST-JANAF thermochemical tables. (1998).
